# The daily association between positive affect and psychotic experiences in individuals along the early stages of the psychosis continuum

**DOI:** 10.3389/fpsyt.2024.1314920

**Published:** 2024-08-29

**Authors:** Sara van der Tuin, Lorna Staines, Larisa Morosan, Esdras Raposo-Almeida, David van den Berg, Sanne H. Booij, Albertine J. Oldehinkel, Johanna T. W. Wigman

**Affiliations:** ^1^ Department of Psychiatry, Interdisciplinary Centre Psychopathology and Emotion Regulation, University of Groningen, University Medical Center Groningen, Groningen, Netherlands; ^2^ Department of Psychiatry, Royal College of Surgeons in Ireland, Dublin, Ireland; ^3^ Institute and Department of Psychiatry (LIM-23), Hospital das Clinicas, School of Medicine, University of Sao Paulo, Sao Paulo, Brazil; ^4^ Department of Clinical Psychology, Amsterdam Public Health Research Institute, Vrije Universiteit Amsterdam, Amsterdam, Netherlands; ^5^ Department of Psychosis Research and Innovation, Parnassia Psychiatric Institute, The Hague, Netherlands; ^6^ Center for Integrative Psychiatry, Lentis, Groningen, Netherlands

**Keywords:** intensive longitudinal data, protective factors, clinical staging, multilevel modeling, diary data

## Abstract

**Introduction:**

Psychosis often develops gradually along a continuum of severity. Little is known about the role of protective factors such as positive affect (PA) in the development of psychotic experiences (PEs). This study investigated i) the temporal (between-day) and contemporaneous (within-day) daily associations between PA and PEs in individuals at different early clinical stages for psychosis and ii) whether these associations differed per clinical stage.

**Methods:**

Daily diary data for 90 days came from 96 individuals at risk for psychosis, distributed over four subgroups defined according to the clinical staging model (stages 0–1b). We constructed multilevel models with PA as a predictor of PEs and vice versa. We investigated within- and between-person temporal and contemporaneous associations and tested whether these associations differed among early stages with multilevel moderation analyses.

**Results:**

We found no within-person temporal effects between PA and PEs in either direction. Contemporaneously, current-day PA predicted current-day PEs (B = −0.14, *p* < 0.001) and vice versa (B = −0.61, *p* < 0.001). Between persons, more 90-day PA predicted fewer PEs in the temporal model (B = −0.14, *p* = 0.03). In addition, more 90-day PEs predicted PA in the temporal (B = −0.26, *p* < 0.001) and contemporaneous (B = −0.36, *p* < 0.001) models. The contemporaneous association between PA and PEs was stronger in individuals at ultra-high risk (UHR) for psychosis than in earlier stages.

**Discussion:**

Our study supported a significant within-day, bidirectional relationship between PA and PEs. This suggests that a focus on PA and methods to improve PA may be an important addition to early intervention practices, particularly in those at UHR for psychosis.

## 1 Introduction

The psychosis continuum proposes that all psychotic phenomena (such as hallucinations, delusions, and paranoia) exist and develop along a continuum of severity (1, 2). On one end of this continuum are mild, infrequent events, referred to as psychotic experiences (PEs), and at the other end are clinical psychotic symptoms (3, 4). Once developed, psychotic disorders are associated with adverse outcomes including higher mortality rates and death by suicide (5), hospitalization (6), and poor long-term outcomes (7, 8). Intervening at an early stage, i.e., *before* a psychotic disorder develops, is therefore of great importance. Early intervention is accommodated by the clinical staging model that identifies stages prior to a clinical psychotic disorder (9). These clinical stages range from stage 0 (increased psychometric or genetic risk) to stage 4 (severe and unremitting illness). Although the stages per definition differ in terms of symptom manifestation, it remains an open question whether factors and mechanisms that contribute to, or may halt, the development of psychosis also differ per clinical stage.

A substantial number of studies focused on risk factors for PEs (for reviews, see (1, 10)) that may induce movement toward more severe parts of the continuum. Fewer studies, however, have examined factors that may halt, prevent, or perhaps even reverse this process (3, 11). Such factors can be seen as protective factors and may be equally important to investigate (12). Ignoring these protective factors misses a key component of mental functioning and thus does not do justice to the full spectrum of mental health (12). Including protective factors in research is critical to advance our knowledge on how such factors are associated with—and may positively contribute to—mental illness in general and psychosis in particular (13). Finding protective factors that positively impact PEs may help in determining additional targets in early intervention services for those at risk of developing psychosis.

Previously identified protective factors for PEs are primarily social protective factors, that is, protective factors that exist in relationship with one’s direct environment. These include having a partner or pet (14), perceived social support (15–18), parental support (19, 20), and living in a socially cohesive community (17, 21). Several person-specific protective factors, that is, protective factors that are intrinsic to the individual, have also been identified. These include personality traits of low neuroticism; higher openness, conscientiousness, agreeableness, and extraversion (14); higher IQ (17, 21); resilience (22, 23); positive characteristics; and social skills (24).

Another protective factor of interest is positive affect (PA). Attempting to define PA to a more specific understanding than it being a measure of positive feelings (25) has proved challenging (26). Some research treats PA and positive emotions as interchangeable (25), while other work more specifically focuses on PA as being associated with emotional reactivity (27). A newer model is the 12-point circumplex structure of “core affect” which focuses on a multi-dimensional approach to understanding affect (26). Core affect does not treat affect and emotions as interchangeable, although core affect is viewed as a component of emotional states. Within the context of this paper, we focus on the “positive” components of the core affect circumplex structure. Jongeneel et al. (28) found that daily PA was negatively associated with daily voice hearing in a sample of individuals with persistent, frequent, and distressing auditory verbal hallucinations. In addition, several studies showed that having psychotic symptoms is associated with less PA in clinical samples (29, 30). Having psychotic experiences is also known to negatively impact wellbeing (31). However, little is known about the mechanisms underlying the interaction between PA and PEs, in terms of, e.g., timing or directionality of this association. One way to examine this is using intensive longitudinal data to investigate this interaction in daily life. In designs collecting such data, one’s PEs and PA are measured multiple times, providing the ability to examine daily associations within an individual. Despite the growing body of intensive longitudinal data in mental health research (32), there are still significant gaps in our knowledge of how PEs and protective factors, particularly personal protective factors such as PA, are associated. In addition, most research into the association between PA and PEs has focused on individuals with diagnosed psychosis. Assessing the nature of this association in the early at-risk stages and whether associations are consistent across early stages may shine new light on whether incorporating PA in early stages can improve outcomes.

This study set out to address the current knowledge gap on protective factors by investigating how PA is related to PEs. First, we examined the bidirectional relationship between daily reports of PA and PEs in daily life in individuals at different levels of risk for psychosis (i.e., in early clinical stages). We assessed both temporal (between-day) and contemporaneous (within-day) associations and within- and between-individual associations. Within-individual associations show how daily fluctuations in one variable are associated with daily fluctuations in another symptom in each individual. Between-individual associations show whether general (i.e., over the complete diary period) levels of one variable are associated with daily levels of the other variable across individuals. The added value of the between-individual associations is to see whether there is also a more trait-like association between PA and PE in addition to assessing day-to-day associations. Second, we aimed to examine whether the temporal and contemporaneous associations between PA and PEs varied per clinical stage.

We hypothesized that within individuals, higher levels of PA are bidirectionally associated with lower PEs, both temporal and contemporaneous. In addition, between individuals, we hypothesized that higher levels of PA, in general, are associated with lower levels of daily PE and, vice versa, that higher levels of PEs, in general, are associated with lower daily PA. We expected differences in the association between PA and PEs across different clinical stages, in line with previous research (33).

## 2 Materials and methods

### 2.1 Participants and study design

**Figure 1:**
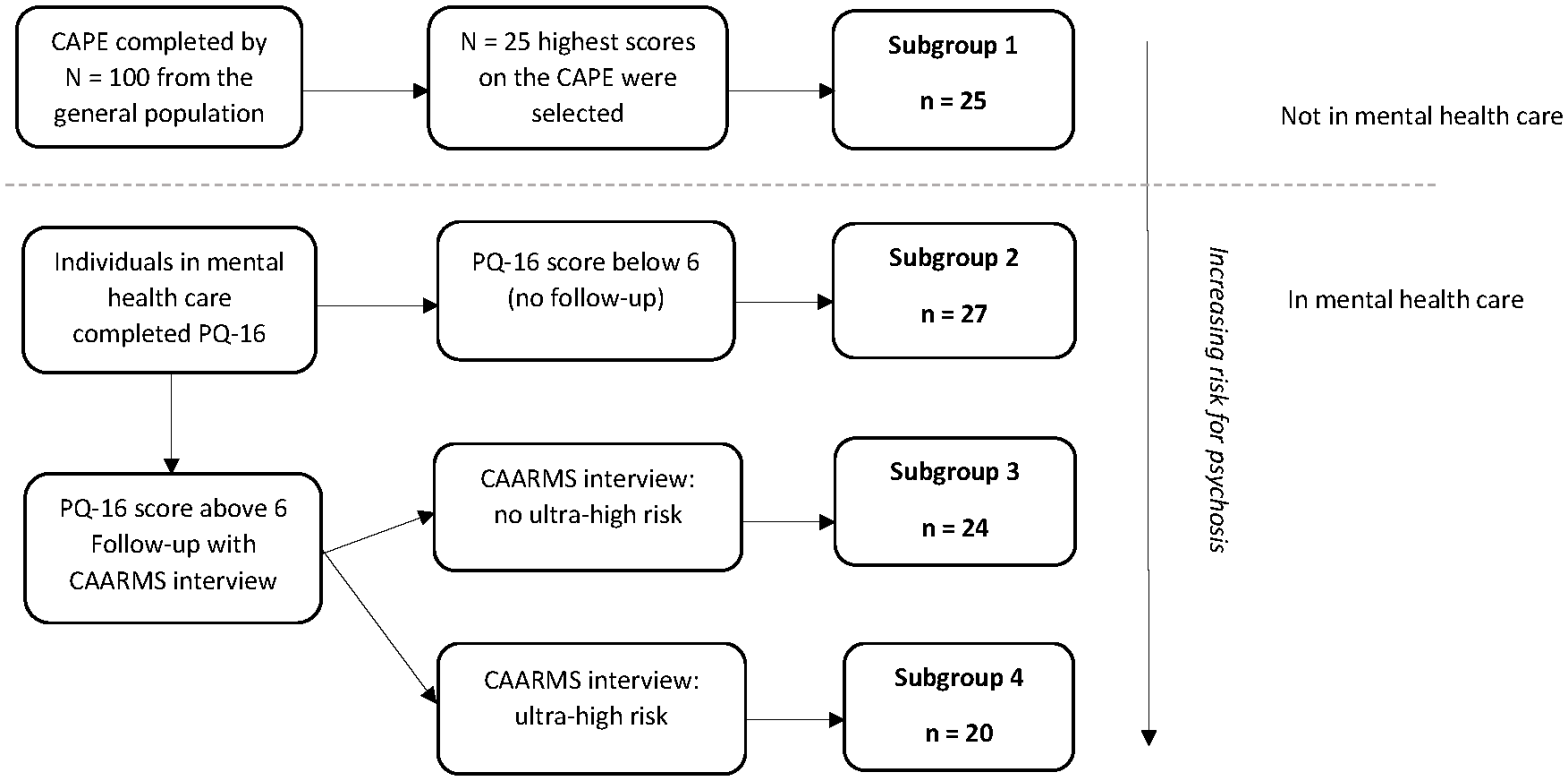
Allocation of individuals to subgroups.

Data from the *Mapping Individual Routes of Risk and Resilience* (Mirorr) study were used. Mirorr is a daily diary study with three yearly follow-up measurements on a broad range of outcomes on mental health and functioning. Mirorr contains four subgroups of individuals in the early clinical stages of psychosis based on the first stages of the clinical staging model for psychosis (9) with an additional distinction between low and minor PEs in individuals in stage 1a. Subgroup 1 consists of individuals from the general population with a relatively high level of psychotic experiences (stage 0); i.e., these individuals are considered at psychometric risk for psychosis (32). These individuals had the 25% highest scores on a questionnaire assessing PEs from a group of 100 individuals from the general population. Individuals in subgroups 2–4 were in mental healthcare and were allocated to specific subgroups based on their level of psychotic experiences [subgroup 2 reflecting stage 1a with low PEs, subgroup 3 reflecting stage 1a with mild PEs, and subgroup 4 reflecting stage 1b (Ultra High Risk for psychosis); see **Figure 1**]. Inclusion criteria were as follows: age between 18 and 35 years, ability to read and speak Dutch fluently and to follow the research procedures, and provided informed consent. Exclusion criteria were as follows: a history of/or current psychotic episodes according to the Diagnostic and Statistical Manual of Mental Disorders, 4th edition (DSM-4) criteria, significant hearing or visual problem impairments, and pregnancy.

For the current study, we used baseline daily diary data in which individuals received 80 items on their smartphone each evening for 90 subsequent days. The diary items were based on previous diary studies and cross-sectional questionnaires and, when necessary, adapted for daily use (34–36). The 80 items concern a broad range of transdiagnostic feelings and experiences that are typical for subclinical and clinical psychosis, depression, anxiety, mania, obsessive-compulsive behavior, and anger. In addition, we used baseline questionnaire data to describe the sample and the subgroups in more detail. For more information about the participants, items, design, and procedure, see Booij et al. (37); for details on the baseline assessments, see Wigman et al. (33).

The study was approved by the medical ethical committee of the University Medical Centre Groningen, Groningen, the Netherlands (registration number MEC no. 2015/159, ABR no. NL52974.042.15). The study was conducted in accordance with the Declaration of Helsinki. All participants provided written informed consent.

### 2.2 Measures

#### 2.2.1 Demographic characterization

The four subgroups and the total sample, age, gender, and education level are described in **Table 1**.

**Table 1:** Descriptive statistics per subgroup and for the total sample.

	Subgroup 1 N = 25	Subgroup 2 N = 27	Subgroup 3 N = 24	Subgroup 4 N = 20	Total group N = 96	Difference^*^
Cross-sectional measurements						
*Demographics*						
Age, mean (SD)	23.3 (3.38)	24.8 (3.95)	26.1 (4.12)	24.8 (5.28)	24.7 (4.20)	ns
Gender (% female)	80.0	74.1	70.8	80.0	76.0	ns
Completed education^1^						
Low (%)	4.0	18.5	8.3	30.0	14.5	ns
Middle (%)	56.0	51.9	58.3	50.0	54.2	ns
High (%)	40.0	25.9	29.2	20.0	29.2	ns
Other (%)	0	4.0	4.0	0	2.1	
*Clinical functioning*						
SCL-90 mean (SD)	141.44 (38.24)	173.78 (45.08)	211.04 (56.09)	232.50 (57.29)	186.91 (59.34)	4, 3, 2 > 1; 4, 3 > 2
Diary items per domain						
Positive affect mean (SD)	57.72 (11.15)	44.68 (12.93)	45.50 (14.18)	43.75 (11.31)	48.09 (13.59)	1 > 2, 3, 4
Psychotic experiences median (IQR)	6.40 (3.74)	6.56 (6.67)	6.98 (11.27)	22.92 (14.90)	7.83 (9.68)	4 > 1, 2, 3; 3 > 1

For psychotic experiences, we noted the median and IQR, as the distribution was skewed.

SCL-90, Symptom Checklist-90-Revised; IQR, interquartile range.

^*^Significant difference p < 0.05, ns, not significant.

^1^Low, primary education or lower secondary education; Medium, upper secondary education, and High, university/college education.

#### 2.2.2 General psychopathology

General psychopathology was measured using the Dutch Symptom Checklist-90-Revised (SCL-90) (38). The SCL-90 consists of 90 questions about different areas of psychopathology. The SCL-90 has high reliability (ω = 0.98) (39) and excellent internal consistency in our sample (Cronbach’s alpha = 0.98).

#### 2.2.2 Diary measures

##### 2.2.2.1 Positive affect

PA comprised of the following six items: “I felt relaxed today”, “I felt calm today”, “I felt satisfied today”, “I felt energetic today”, “I felt enthusiastic today”, and “I felt cheerful today”. The six items for positive affect were based on the 12-point circumplex model of affect (26). Each item was scored on a 0–100 Visual Analog Scale (VAS). A mean PA score per day was calculated by averaging the six items. We calculated a person-mean (PM) score by averaging each individual’s scores over 90 days. In addition, we calculated a person-mean-centered (PMC) score by subtracting each daily score from this person-mean; hence, the PMC reflects individuals’ daily variations in PA around their person-centered means. To assess whether the PA items loaded on the same factor, we calculated the within- and between-level omega using the “omegaSEM” function from the multilevelTools (40) package. This measure of composite reliability takes the multilevel structure of the data into account (41). The within-level omega was 0.89, and the between-level omega was 0.98.

##### 2.2.2.2 Psychotic experiences

PEs comprised of the following five items: “I felt suspicious today”, “Today I had the feeling others disliked me”, “I felt that others could read my thoughts today”, “I felt unreal today”, and “I felt that others could control me today”. These items focus mainly on paranoid ideas and bizarre experiences. While we also assessed the experience of hallucinations, we excluded these items for the current analyses, as they were rarely endorsed, making the data highly skewed and adding little to no information to the analyses. We scored each item on a 0–100 VAS. We calculated a total PE score per day by averaging the five items. We created PM and PMC values and calculated the composite reliability identical to the approach for PA. The within-level omega was 0.60, and the between-level omega was 0.90.

### 2.3 Statistical analyses

All analyses were performed in R version 4.1.3 (42). All multilevel models were created in the R-package “nlme” (43). An alpha of 0.05 was used as an inference criterion.

#### 2.3.1 Descriptive analyses

We calculated age, gender, education level, psychopathology, and daily PA and PE per subgroup and for the total sample. For daily PA, we reported the mean, and for daily PE, we reported the median; for the latter, the distribution was skewed. For the diary data, we first calculated the within-person mean or median before calculating the mean or median of the subgroups and the total sample. We compared the subgroups with each other on the SCL-90 by means of an ANOVA. In addition, we compared the subgroups to each other on daily PA and PE by means of two multilevel models.

#### 2.3.2 Bidirectional temporal and contemporaneous relationships between PA and PE

We constructed four multilevel models to assess the bidirectional daily association between PA and PE: two temporal (between-day) models and two contemporaneous (within-day) models. In the temporal models, we assessed the association between PA from the previous day (t − 1) on PE from the current day (t) and vice versa. In the contemporaneous models, we assessed the within-day association between PA (t) and PE (t). Please note that although no temporal order can be determined within days, we constructed the contemporaneous models in both directions [i.e., PA (t) predicting PE (t) and vice versa]. The main difference between these two models was the addition of the lagged (t − 1) value of the outcome to control for autoregressive effects. In all models, we used the PMC and PM values of the predictor, as well as the lagged value (t − 1) of the outcome, to control for autocorrelation. We added time (days 1–90, centered around the mean) as a covariate to control for linear trends in the data and a random intercept for individuals to account for individual variation.

We checked whether adding random slopes of the predictors improved the model by assessing the Akaike information criterion (AIC). A lower AIC indicates a better model. For both temporal models (models 1 and 2), only including the random slope of the lagged value of the outcome provided the best model fit. For both contemporaneous models (models 3 and 4), including random slopes of both the lagged value of the outcome and the PMC value of the predictor provided the best model fit. Second, we assessed which covariance matrix performed best—Natural, Diagonal, Compound Symmetry, or Identity—based on the AIC. For all models, except model 1, the best covariance structure was “natural”. The best covariance structure for model 1 was diagonal, but the difference between the covariance structure “natural” and “diagonal” was minimal (covariance matrix natural AIC = 50,537.31 and diagonal AIC = 50,536.85). For consistency between all models, we, therefore, chose the covariance matrix “natural” for all models.

For all moderation analyses, we used the covariance matrix “natural”, as this had the best model fit. Testing of random effects and covariance structure was performed using the method Restricted Maximum Likelihood (REML), as that provides the most accurate estimates for random effects. As our final goal was to compare fixed effects, all final models were run with Maximum Likelihood (ML), which estimates the fixed effects best.

#### 2.3.3 Subgroup differences in the association between PA and PE

To assess whether the subgroup moderated the association between PA and PE, we added the interaction between the PMC predictor and subgroup as fixed effects to the four models described above. We took the same steps as in the above-described models to assess whether random effects improved model fit and to select the best covariance matrix. We corrected for multiple testing with the False Discovery Rate (FDR) method (44).

#### 2.3.4 Power

In this study, we used multilevel models for which the power is determined by a combination of the number of individuals and the number of observations per individual. For all models, we had 96 individuals with 70–90 measurements per individual. Monte Carlo simulations indicated that with a dataset of 96 individuals with 70 observations per person, a medium effect size (0.30) (45), medium-high inter-class correlation coefficient (ICC) of 0.3 (46), and a medium variance of 0.09 yield a power of 99% to detect an effect. In the moderation analyses, the power to detect an interaction effect was still 99%. Thus, for all analyses, we expected sufficient power to detect relevant effects.

## 3 Results

### 3.1 Sample characteristics

The sociodemographic, clinical, and diary characteristics of the subgroups and total sample are described in **Table 1**. The subgroups did not differ significantly in age, gender, and education level. Individuals in higher subgroups reported, in general, higher psychopathology scores, lower scores on daily PA, and more daily PEs. Subgroup 1 had significantly higher PA scores than the other subgroups, and subgroup 4 had significantly higher PE scores than the other subgroups. Participants had, on average, 8% (range, 0%–22%) missing data for the diary items.

### 3.2 Bidirectional temporal and contemporaneous relationships between PA and PE

The full details of each model are reported in **Table 2**.

**Table 2:** Bidirectional associations between PA and PE (models 1–4) for the total sample.

Fixed effects	B	SE	*p*-Value
*Model 1: PE (t) predicted by PA (t − 1) (temporal)*			
(Intercept)	16.84	3.08	<0.001
PA PMC (t − 1)	−0.01	0.01	0.35
PA PM	−0.14	0.06	0.03*
PE (t − 1)	0.20	0.02	<0.001**
Time	0.00	0.01	0.78
*Model 2: PA (t) predicted by PE (t − 1) (temporal)*			
(Intercept)	40.02	1.70	<0.001
PE PMC (t − 1)	0.01	0.02	0.70
PE PM	−0.26	0.08	<0.001**
PA (t − 1)	0.25	0.02	<0.001**
Time	0.00	0.01	0.98
*Model 3: PE (t) predicted by PA (t) (contemporaneous)*			
(Intercept)	16.28	3.05	<0.001
PA PMC	−0.14	0.02	<0.001**
PA PM	−0.11	0.06	0.06
PE (t − 1)	0.15	0.02	<0.001**
Time	0.01	0.01	0.50
*Model 4: PA (t) predicted by PE (t) (contemporaneous)*			
(Intercept)	43.03	1.72	<0.001
PE PMC	−0.61	0.05	<0.001**
PE PM	−0.36	0.08	<0.001**
PA (t − 1)	0.21	0.02	<0.001**
Time	0.00	0.01	0.93

PE, psychotic experiences; PA, positive affect; PMC, person-mean centered; PM, person-mean.

*p < 0.05, **p < 0.01.

#### 3.2.1 Model 1: PE (t) predicted by PA (t − 1)

Within individuals, PMC PA did not significantly predict PEs the following day. Between individuals, PM PA was significantly associated with lower levels of daily PEs. PEs (t − 1) significantly predicted PEs (t), indicating a positive autoregressive effect of PEs.

#### 3.2.2 Model 2: PA (t) predicted by PE (t − 1)

Within individuals, PMC PEs (t − 1) did not predict PA (t). Between individuals, PM PE was significantly associated with lower levels of daily PA. PA (t − 1) significantly predicted PA (t), indicating a positive autoregressive effect.

#### 3.2.3 Model 3: PE (t) predicted by PA (t)

Within individuals, PMC PA (t) significantly predicted PEs (t). Between individuals, PM PA was not significantly associated with lower daily PEs (t). There was a positive autoregressive effect of PEs.

#### 3.2.4 Model 4: PA (t) predicted by PE (t)

Within individuals, PMC PEs (t) significantly predicted PA (t). Between individuals, PM PEs were associated with lower daily PA (t). There was a positive autoregressive effect of PA.

### 3.3 Moderation analyses

**Figure 2:**
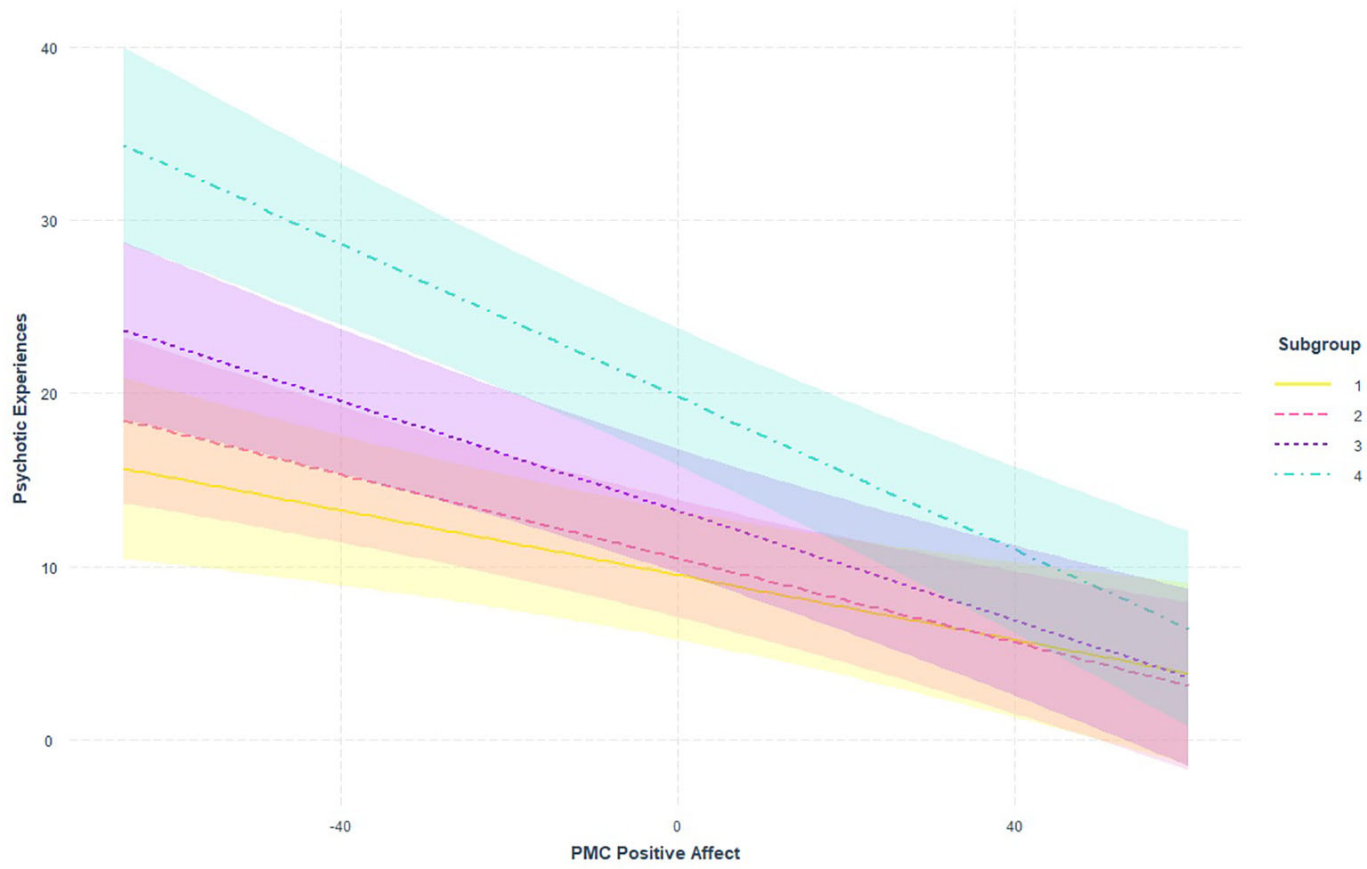
Visualization of the moderating effect of subgroup on the within-day association between PA and PE from model 3 (PE predicted by PA during the same day). PA, positive affect; PE, psychotic experience.

We found two significant moderation effects of the subgroup on the contemporaneous (within-day) effect of PA on PE: subgroup 4 had stronger (negative) associations than subgroup 1 (B = −0.13, *p* = 0.003) and subgroup 2 (B = −0.10, *p* = 0.02) (see **Supplementary Tables 1**-**4** for the results of the moderation analyses). This indicates that the within-day effect of PA on PE is stronger in individuals at ultra-high risk (UHR) for psychosis (stage 1b) than in individuals at psychometric risk for psychosis (stage 0) and those with low PEs (stage 1a). After correcting for multiple testing with FDR, both differences remained significant (subgroup 4 − subgroup 1: *p* = 0.01; subgroup 4 − subgroup 2: *p* = 0.049). The results are visualized in **Figure 2**. This showed that groups differed mostly in PEs at low PA levels. We found no significant moderation effects in the other models.

## 4 Discussion

This study investigated the bidirectional, temporal (between-day), and contemporaneous (within-day) associations between PA and PEs. We also examined whether these associations varied across the early clinical stages of psychosis. We observed no within-individual temporal associations between PE and PA in either direction. Regarding the contemporaneous within-individual associations, daily fluctuations in PA were associated with daily fluctuations in PEs. In addition, between individuals, those who generally reported more PA reported fewer daily PEs and vice versa. We found a stronger within-individual contemporaneous association between PA and PEs in individuals at UHR for psychosis (stage 1b; subgroup 4), compared to individuals from the general population at increased psychometric risk (subgroup 1, stage 0) and individuals in mental healthcare with low PEs (subgroup 2, stage 1a). Thus, PA and PEs were negatively associated at both within- and between-person levels.

With our daily measures, we did not find a day-to-day association between PA and PEs. Our study suggests that daily-life PA does not have a long-lasting (24-hour+) protective effect against PEs and that PEs do not have a long-lasting adverse effect on subsequent daily-life PA. While it is possible that there is no temporal effect of PA on PEs and vice versa, we believe it is premature to conclude this. Rather, the explanation can lie in the sampling frequency in our study. To the best of our knowledge, this is the first study that investigated the daily association between PA and PEs across different early clinical stages of psychosis. Still, several studies have used intensive longitudinal data to look at associations between other protective factors and PEs. Both Thewissen et al. (47) and Monsonet et al. (13) found a temporal association between self-esteem and PEs, and they had a much higher sampling rate of respectively 10 and 8 times per day, as compared to our study. Therefore, these temporal associations in fact may reflect within-day associations, which align with the contemporaneous association between PA and PE that we found. In addition, Jongeneel et al. (28) also found only within-day associations between PA and voice hearing. As we did not find between-day (temporal) associations, it is possible that the rate at which protective factors act on PEs is higher than measured in our study, i.e., in hours not days. The time scale on which mechanisms underlying psychopathology and its interaction with protective and risk factors play out is currently unknown (48), and thus, it is not clear which sampling rate best reflects this time scale. It is likely to be expected that this optimal time frame differs per symptom (e.g., mood versus hearing voices) and per risk or protective factor (e.g., self-esteem versus optimism).

We assessed the contemporaneous association in both directions, but it is important to keep in mind that we cannot draw conclusions on directionality, as PA and PE were measured at the same moment. As may be expected, we found largely comparable results in both directions, with some minor differences, which can be explained by the inclusion of different covariates in the models. Regardless of these differences, both models led to the same conclusion: within persons, there is a negative within-day association between PA and PEs. In addition to these within-person effects, we found that between individuals, having higher PA over 90 days was associated with fewer PEs and vice versa. This implies that PA and PE are negatively associated both within and between individuals.

There are several hypothetical mechanisms that could explain any association between PA and PE that may play out at a smaller time scale. High levels of PA may impact PE in various ways. First, PEs are most likely to persist and have a negative impact when distressing (49). High levels of PA may counter the distressing nature of PEs and, consequently, their potentially negative impact. This mechanism can be understood in the context of the broaden-and-build theory (50). This theory, with its basis in positive psychology, posits that positive emotions broaden an individual’s momentary thought-action repertoires and build enduring personal resources (50). In this way, increases in PA may increase personal resources that may act as a buffer to PEs. Second, high levels of PA facilitate approach behavior, i.e., make an individual more likely to actively engage with their environment (51, 52). As such, high PA may stimulate individuals with PEs to actively seek out social contact to discuss their thoughts or behavior. This social interaction may contribute to challenging or countering PEs, such as paranoid thoughts or false (delusional) beliefs, resulting in the alleviation of the PE or its impact.

However, PEs can negatively impact PA as well in several ways; PEs can be distressing in nature and thereby decrease levels of PA (31, 49). In addition, PEs can impact other life domains as well. For example, having PEs can lead to less social support (53), which likely negatively impacts PA. As we cannot draw any conclusions about directionality in our study, more research is necessary to assess the directionality of the association between PA and PEs. We recommend future research to use a higher sampling rate (e.g., five to seven times a day), as it is possible that the process in which PA and PE fluctuate and interact is more rapid than the daily time frame we investigated in our study. Finding the directionality of the association between PA and PE would have potential clinical implications. Boosting PA in individuals could be investigated as an additional target to improve treatment outcomes.

Moderation analyses indicated that the contemporaneous association between PA and PEs was stronger in individuals at UHR for psychosis than in those with lower levels of PEs. This is in contrast to Monsonet et al. (13), who found that protective factors acted the same across a schizotypy continuum that included trait, subclinical, clinical risk, and overt clinical expressions. One explanation for this incongruence may lie in the specific protective factor that was assessed. Monsonet et al. (13) investigated the daily association between self-esteem and PEs, whereas we focused on PA. Our finding of a stronger association between PEs and PA in those at UHR for psychosis was in line with our hypothesis. One possible explanation is not only did individuals in the UHR group experience more frequent and severe PEs but also that the content and/or distressing nature of their PEs differs qualitatively from those in lower clinical stages (9, 54). Further research using qualitative methods would help to further understand this interaction.

Our results should be considered in the light of several strengths and limitations. This is, to the best of our knowledge, the first study to directly assess the daily association between PA and PEs in individuals in the early clinical stages of psychosis. With a long measurement period of 90 days, we were also able to capture both PA and PEs once a day over a longer period. This is likely to be more representative of daily life than a shorter period, which is more easily influenced by specific events. We only had 8% missing data on average, and the completion time for the diary was 5 minutes on average, making the burden on participants relatively low and therefore giving us confidence that participants were able to complete the diaries with attention. That said, this design also has drawbacks. In our study, we choose a relatively low sampling rate of one measurement per day. While this has the advantages of allowing a longer measurement period and picking up fewer frequent events (such as subclinical psychotic experiences), it also has the limitation that we cannot draw any conclusions about any association between PA and PE that may unfold over a shorter time period. Because we only found within-day associations, we cannot draw conclusions on the directionality of the association between PA and PE. To investigate this in more detail, our study should be replicated using a design of multiple measurements per day. Furthermore, we did not have sufficiently detailed information on treatment and medication and therefore could not take this into account in the analyses. It cannot be ruled out that some individuals already received treatment focusing (partly) on increasing PA. Another limitation is that our measure of PEs did not encompass all forms of PEs but mainly paranoid ideas and bizarre experiences. We deliberately excluded hallucinations from the PE measure, as these were very rarely endorsed in our sample (33), which made the data highly skewed and would add little information to our analyses. Consequently, we do not know whether the associations found hold for hallucinations as well. Jongeneel et al. (28) did find a within-day association between voice hearing and PA and thus provide preliminary evidence for an association between hallucinations and PA.

### 4.1 Conclusion

Our results showed that within individuals, PA and PE were negatively associated within days. We did not find this association between days; i.e., PA on the previous day did not predict PEs on the current day, or vice versa. We found that the contemporaneous within-person association was stronger in individuals at UHR for psychosis than in individuals in earlier clinical stages. In addition, between individuals, we found that individuals who overall had higher levels of PA experienced fewer PEs on a daily basis and vice versa. Taken together, our results highlight the importance of including protective factors like PA in studying PE development in individuals in the early clinical stages of psychosis, especially in those at UHR for psychosis. Future research should focus on further elucidating the temporal association between PA and PEs, the association between PA and PEs in other clinical stages, and the buffering role of other protective factors.

## Data Availability

The raw data supporting the conclusions of this article will be made available by the authors, without undue reservation.

## Supplementary material

Supplementary Table 1 Moderation effects PA (t-1) predicting PE (t) PE, psychotic experiences, PA, positive affect, PMC, person-mean centered, PM, person-mean, **p* <.05, ***p* <.01.

Supplementary Table 2 Moderation effects PE (t-1) predicting PA (t) PE, psychotic experiences, PA, positive affect, PMC, person-mean centered, PM, person-mean, **p* <.05, ***p* <.01.

Supplementary Table 3 Moderation effects PA (t) predicting PE (t) PE, psychotic experiences, PA, positive affect, PMC, person-mean centered, PM, person-mean, **p* <.05, ***p* <.01.

Supplementary Table 4 Moderation effects PE (t) predicting PA (t) PE, psychotic experiences, PA, positive affect, PMC, person-mean centered, PM, person-mean, **p* <.05, ***p* <.01.
