# Supplementary material for: The daily association between positive affect and psychotic experiences in individuals along the early stages of the psychosis continuum
Source: Front Psychiatry. 2024 Aug 29;15:1314920. doi: 10.3389/fpsyt.2024.1314920 (PMC11390539; doi:10.3389/fpsyt.2024.1314920)
Supplement: Supplementary Table 1 — Moderation effects PA (t-1) predicting PE (t) PE, psychotic experiences, PA, positive affect, PMC, person-mean centered, PM, person-mean, *p <.05, **p <.01. [file Table1.docx]

**Supplementary Table 1: Moderation effects PA (t-1) predicting PE (t)**

| **Fixed effect** | **B** | **SE** | **p-value** |
| --- | --- | --- | --- |
| *Reference group: subgroup 1* | | | |
| (Intercept) | 10.22 | 3.70 | 0.01 |
| PA PMC (t-1) | -0.01 | 0.01 | 0.64 |
| PA PM | -0.07 | 0.06 | 0.25 |
| PE (t-1) | 0.20 | 0.02 | 0.00 |
| Time | 0.00 | 0.01 | 0.81 |
| Subgroup2 | 0.77 | 2.08 | 0.71 |
| Subgroup3 | 3.66 | 2.11 | 0.09 |
| Subgroup4 | 10.45 | 2.27 | 0.00 |
| PA PMC (t-1):subgroup2 | 0.01 | 0.02 | 0.59 |
| PA PMC (t-1):subgroup3 | -0.02 | 0.02 | 0.28 |
| PA PMC (t-1):subgroup4 | 0.01 | 0.02 | 0.58 |
| *Reference group: subgroup 2* | | | |
| (Intercept) | 10.98 | 2.99 | 0.00 |
| PA PMC (t-1) | 0.00 | 0.01 | 0.79 |
| PA PM | -0.07 | 0.06 | 0.25 |
| PE (t-1) | 0.20 | 0.02 | 0.00 |
| Time | 0.00 | 0.01 | 0.81 |
| Subgroup1 | -0.77 | 2.08 | 0.71 |
| Subgroup3 | 2.89 | 1.96 | 0.14 |
| Subgroup4 | 9.68 | 2.08 | 0.00 |
| PA PMC (t-1):subgroup1 | -0.01 | 0.02 | 0.59 |
| PA PMC (t-1):subgroup3 | -0.03 | 0.02 | 0.09 |
| PA PMC (t-1):subgroup4 | 0.00 | 0.02 | 0.94 |
| *Reference group: subgroup 3* | | | |
| (Intercept) | 13.87 | 3.10 | 0.00 |
| PA PMC (t-1) | -0.03 | 0.01 | 0.04 |
| PA PM | -0.07 | 0.06 | 0.25 |
| PE (t-1) | 0.20 | 0.02 | 0.00 |
| Time | 0.00 | 0.01 | 0.81 |
| Subgroup1 | -3.66 | 2.11 | 0.09 |
| Subgroup2 | -2.89 | 1.96 | 0.14 |
| Subgroup4 | 6.79 | 2.15 | 0.00 |
| PA PMC (t-1):subgroup1 | 0.02 | 0.02 | 0.28 |
| PA PMC (t-1):subgroup2 | 0.03 | 0.02 | 0.09 |
| PA PMC (t-1):subgroup4 | 0.03 | 0.02 | 0.11 |

PE = psychotic experiences, PA = positive affect, PMC = person-mean centered, PM = person-mean, **p* < .05, ***p* < .01

**Supplementary Table 2: moderation effects PE (t-1) predicting PA (t)**

| **Fixed effect** | **B** | **SE** | **p-value** |
| --- | --- | --- | --- |
| *Reference group: subgroup 1* | | | |
| (Intercept) | 44.97 | 2.03 | 0.00 |
| PE PMC (t-1) | -0.03 | 0.06 | 0.68 |
| PE PM | -0.18 | 0.08 | 0.03 |
| PA (t-1) | 0.25 | 0.02 | 0.00 |
| Time | 0.00 | 0.01 | 0.99 |
| Subgroup2 | -9.60 | 2.26 | 0.00 |
| Subgroup3 | -6.91 | 2.37 | 0.00 |
| Subgroup4 | -7.97 | 2.72 | 0.00 |
| PE PMC (t-1):subgroup2 | 0.05 | 0.08 | 0.54 |
| PE PMC (t-1):subgroup3 | 0.02 | 0.07 | 0.77 |
| PE PMC (t-1):subgroup4 | 0.05 | 0.07 | 0.45 |
| *Reference group: subgroup 2* | | | |
| (Intercept) | 44.97 | 2.03 | 0.00 |
| PE PMC (t-1) | -0.03 | 0.06 | 0.68 |
| PE PM | -0.18 | 0.08 | 0.03 |
| PA (t-1) | 0.25 | 0.02 | 0.00 |
| Time | 0.00 | 0.01 | 0.99 |
| Subgroup2 | -9.60 | 2.26 | 0.00 |
| Subgroup3 | -6.91 | 2.37 | 0.00 |
| Subgroup4 | -7.97 | 2.72 | 0.00 |
| PE PMC (t-1):subgroup1 | 0.05 | 0.08 | 0.54 |
| PE PMC (t-1):subgroup3 | 0.02 | 0.07 | 0.77 |
| PE PMC (t-1):subgroup4 | 0.05 | 0.07 | 0.45 |
| *Reference group: subgroup 3* | | | |
| (Intercept) | 44.97 | 2.03 | 0.00 |
| PE PMC (t-1) | -0.03 | 0.06 | 0.68 |
| PE PM | -0.18 | 0.08 | 0.03 |
| PA (t-1) | 0.25 | 0.02 | 0.00 |
| Time | 0.00 | 0.01 | 0.99 |
| Subgroup2 | -9.60 | 2.26 | 0.00 |
| Subgroup3 | -6.91 | 2.37 | 0.00 |
| Subgroup4 | -7.97 | 2.72 | 0.00 |
| PE PMC (t-1):subgroup1 | 0.05 | 0.08 | 0.54 |
| PE PMC (t-1):subgroup2 | 0.02 | 0.07 | 0.77 |
| PE PMC (t-1):subgroup4 | 0.05 | 0.07 | 0.45 |

PE = psychotic experiences, PA = positive affect, PMC = person-mean centered, PM = person-mean, **p* < .05, ***p* < .01

**Supplementary Table 3: moderation effects PA (t) predicting PE (t)**

| **Fixed effect** | **B** | **SE** | **p-value** |
| --- | --- | --- | --- |
| *Reference group: subgroup 1* | | | |
| (Intercept) | 10.45 | 3.72 | 0.01 |
| PA PMC | -0.09 | 0.03 | 0.00 |
| PA PM | -0.06 | 0.06 | 0.31 |
| PE (t-1) | 0.15 | 0.02 | 0.00 |
| Time | 0.01 | 0.01 | 0.51 |
| Subgroup2 | 0.73 | 2.09 | 0.73 |
| Subgroup3 | 3.51 | 2.12 | 0.10 |
| Subgroup4 | 10.38 | 2.28 | 0.00 |
| PA PMC:subgroup2 | -0.03 | 0.04 | 0.48 |
| PA PMC:subgroup3 | -0.07 | 0.04 | 0.11 |
| PA PMC:subgroup4 | -0.13 | 0.04 | 0.003* |
| *Reference group: subgroup 2* | | | |
| (Intercept) | 11.18 | 3.01 | 0.00 |
| PA PMC | -0.12 | 0.03 | 0.00 |
| PA PM | -0.06 | 0.06 | 0.31 |
| PE (t-1) | 0.15 | 0.02 | 0.00 |
| Time | 0.01 | 0.01 | 0.51 |
| Subgroup1 | -0.73 | 2.09 | 0.73 |
| Subgroup3 | 2.77 | 1.97 | 0.16 |
| Subgroup4 | 9.65 | 2.09 | 0.00 |
| PA PMC:subgroup1 | 0.03 | 0.04 | 0.48 |
| PA PMC:subgroup3 | -0.04 | 0.04 | 0.34 |
| PA PMC:subgroup4 | -0.10 | 0.04 | 0.016* |
| *Reference group: subgroup 3* | | | |
| (Intercept) | 13.96 | 3.11 | 0.00 |
| PA PMC | -0.16 | 0.03 | 0.00 |
| PA PM | -0.06 | 0.06 | 0.31 |
| PE (t-1) | 0.15 | 0.02 | 0.00 |
| Time | 0.01 | 0.01 | 0.51 |
| Subgroup1 | -3.51 | 2.12 | 0.10 |
| Subgroup2 | -2.77 | 1.97 | 0.16 |
| Subgroup4 | 6.88 | 2.15 | 0.00 |
| PA PMC:subgroup1 | 0.07 | 0.04 | 0.11 |
| PA PMC:subgroup2 | 0.04 | 0.04 | 0.34 |
| PA PMC:subgroup4 | -0.06 | 0.04 | 0.15 |

PE = psychotic experiences, PA = positive affect, PMC = person-mean centered, PM = person-mean, **p* < .05, ***p* < .01

**Supplementary Table 4: moderation effects PE (t) predicting PA (t)**

| **Fixed effect** | **B** | **SE** | **p-value** |
| --- | --- | --- | --- |
| *Reference group: subgroup 1* | | | |
| (Intercept) | 47.61 | 2.11 | 0.00 |
| PE PMC | -0.62 | 0.10 | 0.00 |
| PE PM | -0.26 | 0.09 | 0.00 |
| PA (t-1) | 0.21 | 0.02 | 0.00 |
| Time | 0.00 | 0.01 | 0.92 |
| Subgroup2 | -9.55 | 2.44 | 0.00 |
| Subgroup3 | -6.86 | 2.56 | 0.01 |
| Subgroup4 | -7.29 | 2.93 | 0.01 |
| PE PMC:subgroup2 | -0.03 | 0.14 | 0.82 |
| PE PMC:subgroup3 | -0.03 | 0.14 | 0.84 |
| PE PMC:subgroup4 | 0.12 | 0.14 | 0.39 |
| *Reference group: subgroup 2* | | | |
| (Intercept) | 38.06 | 2.10 | 0.00 |
| PE PMC | -0.65 | 0.09 | 0.00 |
| PE PM | -0.26 | 0.09 | 0.00 |
| PA (t-1) | 0.21 | 0.02 | 0.00 |
| Time | 0.00 | 0.01 | 0.92 |
| Subgroup1 | 9.55 | 2.44 | 0.00 |
| Subgroup3 | 2.69 | 2.50 | 0.28 |
| Subgroup4 | 2.26 | 2.83 | 0.43 |
| PE PMC:subgroup1 | 0.03 | 0.14 | 0.82 |
| PE PMC:subgroup3 | 0.00 | 0.13 | 0.98 |
| PE PMC:subgroup4 | 0.15 | 0.13 | 0.24 |
| *Reference group: subgroup 3* | | | |
| (Intercept) | 47.61 | 2.11 | 0.00 |
| PE PMC | -0.62 | 0.10 | 0.00 |
| PE PM | -0.26 | 0.09 | 0.00 |
| PA (t-1) | 0.21 | 0.02 | 0.00 |
| Time | 0.00 | 0.01 | 0.92 |
| Subgroup2 | -9.55 | 2.44 | 0.00 |
| Subgroup3 | -6.86 | 2.56 | 0.01 |
| Subgroup4 | -7.29 | 2.93 | 0.01 |
| PE PMC:subgroup1 | -0.03 | 0.14 | 0.82 |
| PE PMC:subgroup2 | -0.03 | 0.14 | 0.84 |
| PE PMC:subgroup4 | 0.12 | 0.14 | 0.39 |

PE = psychotic experiences, PA = positive affect, PMC = person-mean centered, PM = person-mean, **p* < .05, ***p* < .01
